# Māori perspectives on sleep and aging

**DOI:** 10.3389/frsle.2024.1410856

**Published:** 2024-06-05

**Authors:** Rosemary Gibson, Hannah Lowe, Erina Korohina, Anna Rolleston

**Affiliations:** ^1^School of Psychology, Massey University, Manawatu, New Zealand; ^2^Manawa Ora, The Centre for Health, Tauranga, New Zealand

**Keywords:** gerontology, grandparenting, group interviews, Indigenous health, New Zealand, rest, routines, spirituality

## Abstract

**Introduction:**

Sleep is vital for health in older adulthood. Ethnic disparities have been noted with regards to sleep health. However, culturally appropriate approaches to sleep as a broader social experience are lacking.

**Methods:**

Here, sleep-related group interviews were conducted in the form of hui (group meetings and discussions) with eleven participants of a health service intervention for older Māori (the Indigenous people of New Zealand) and their whānau (extended family). Notes were collated and analyzed thematically.

**Results:**

Four key themes were constructed that represent the key conversations and ideas. These concerned the conceptualizing of sleep—including appreciation for its somatic role but also the spiritual properties of sleep states; the changing obligations around sleep and wake—including individual and communal time use and changing cultural and familial obligations with advancing age; and the barriers and facilitators for supporting sleep—including the social and spiritual nature of communal sleeping, the schedules and sleep of others, as well as holistic and environmental methods for relaxation. Findings demonstrate the multifaceted nature of sleep and aging among Māori. Culturally relevant interpretations of sleep practices and disturbances were offered and are beyond typical Western models which are predominantly medicalized.

**Discussion:**

This work aids the understanding and representation of sleep as a social and cultural perspective within the New Zealand context. This provides foundations for future participatory research to design culturally appropriate approaches to assessing and supporting sleep health in forms that are meaningful for aging well across cultures.

## 1 Introduction

Sleep has been identified as pivotal for health, functioning, and wellbeing across the life-course (Ramar et al., 2021). Adequate sleep has been associated with improved mood, mental health, cognitive function, and various aspects of physical health such as metabolic, cardiovascular, and cerebrovascular health (Reid et al., 2006; Alvaro et al., 2013). Problematic sleep is considered more prevalent with older adulthood. Physiological deterioration and illness appear to increase the likelihood of sleep-related disorders like insomnia, sleep apnea, or advanced sleep phase (Miner and Kryger, 2020). Such sleep problems have subsequently been independently related to negative outcomes such as frailty, dementia, falls, an earlier need for formal care, as well as earlier mortality (Bliwise, 2016; Gibson et al., 2017; Gibson and Gander, 2020).

It is estimated that over a quarter of adults in Aotearoa, New Zealand (NZ) have sleeping problems (Paine et al., 2005; Gibson et al., 2015, 2023a). Socioeconomic and ethnic disparities have been identified among young and middle-aged groups. Māori (the Indigenous people of NZ) have been recognized as more prone to insufficient sleep or sleep disorders compared to non-Māori (Paine et al., 2005, 2019; Paine and Gander, 2016). This disparity has been attributed to a loss of knowledge through colonization and assimilation processes of Māori values and needs including traditional health and sleeping rituals, as well as the influence of individual and structural racism (Durie, 1985; Slopen and Williams, 2014; Paine and Gander, 2016). Factors like work demands, social schedules, and health behaviors can also negatively affect sleep status (Grandner, 2019). Ethnic disparities have been noted within such factors. For example, Māori have been identified as more likely to work irregular hours or night shifts, and to consume alcohol or smoke compared to non-Māori (Herbert and Stephens, 2015; Paine and Gander, 2016). Despite this, research examining the sleep status of older New Zealanders have been less conclusive. For instance, an analysis of data from Māori and non-Māori individuals aged over 80 years found that fewer Māori participants reported sleep problems (26.3%) compared to non-Māori participants (31.7%) (Gibson et al., 2016, 2020). This contrasts with previous studies involving New Zealanders of retirement age (Gibson et al., 2015). However, when older Māori did report a sleep problem, they corroborated a significantly higher number of symptoms than non-Māori. This potentially reflects unique social and or cultural beliefs, expectations, and perceptions of problematic sleep as well as highlights the limitations of relying on short-form quantitative measures alone (Gibson et al., 2016).

Sleep is typically evaluated with regards to presence or absence of sleep problems. Western scientific perspectives have somewhat colonized and medicalized sleep, leaving little room for alternative interpretations (Williams, 2002, 2011). Therefore, a need for integrated research examining the sociological and cultural elements of sleep has been highlighted (Meadows, 2005; Airhihenbuwa et al., 2016). More holistic approaches are increasingly recognized and applied. Such approaches consider, for example, the dimensions of ‘sleep health' including the duration, quality, timing, and regularity of sleep, as well as alertness during wakefulness which can all be impacted by individual as well as mediating and broader social factors (Buysse, 2014; Grandner, 2019; Hale et al., 2019). Meadows (2005) sociological framework of sleep acknowledges the importance of considering not only the visceral, biological aspects of sleep; but also how sleep is embodied in social norms, the pragmatics of sleep for enabling function and purpose, the sleeper's experience, as well as macro level social and cultural contexts that inform sleep practices. Such a framework is deemed useful for understanding and representing the unique changes to sleep across the lifespan. It also provides a foundation for considering the sleep of Māori who are more likely than Western cultures to live by multi-dimensional and holistic perspectives of health, in which dimensions of taha wairua (spiritual health) and taha taha whānau (extended family) are considered key alongside taha hinengaro (mental health) and taha tinana (physical health). A connection to whenua (land) provides a foundation for these dimensions of wellbeing (Durie, 1985; Valentine et al., 2017; George et al., 2020). Therefore, Māori are considered to be more culturally connected and engaged with obligations with whānau and iwi than non-Māori (McDonald, 2016). This potentially creates tensions aligning with the scheduler nature of work and school (and therefore sleep) in modern capitalist society (Wolf-Meyer, 2019). Time use during retirement is also likely to reflect such beliefs, practices and obligations compared to the more individualistic or scheduled nature of activities among European retirees (Wanka, 2020).

Interviews with healthy older Māori and non-Māori highlighted the importance of individual and social experiences as well as cultural interpretations relating to sleep with aging (Crestani et al., 2022). Crestani et al. (2022) found that participants often compared their sleep to social norms, with deterioration of sleep considered as a gradual and acceptable part of aging. However, subtle cultural differences were also noted. Older Māori interviewees appeared to be less prone to pathologize sleep disruptions than non-Māori. Instead, some spoke of periods of sleeplessness as opportunities for positive cultural, familial, or spiritual connection (Crestani et al., 2022). Such concepts warrant further exploration and representation given the social drivers to identify and manage “problematic sleep.”

Sleep-related research from NZ corroborates international works concerning the importance of sleep for overall health status (Reid et al., 2006; Alvaro et al., 2013). However, also suggests that how sleep problems are understood and reported may vary with aging and between New Zealand's unique social and cultural contexts. Crestani et al. (2022) research was limited to one-on-one interviews with healthy older adults, as opposed to those who had experienced specific health or sleep problems. The aim of the present research was to further explore how sleep as an experience and practice changes as we age among older Māori and their whānau. This was achieved using a hui approach (group meetings and discussions) to facilitate open conversations around the functions and regulation of sleep and its alignment with individual and whānau values, as well as beliefs and practices concerning sleep problems and their management incorporating Māori worldviews.

## 2 Methods

### 2.1 Group interviews

The approach to this work honors and utilizes the background and experiences of the research team particularly with regards to their whakapapa and mātauranga Māori (Māori ancestry and knowledge AR & EK); experience with clinical health care (HL & AR) and sleep psychology (RG). Group interviews were established in the form of a hui providing a space for group-based knowledge sharing and production as well as open discussion and storytelling. All tikanga (correct procedures, customs) and cultural practices were maintained by Manawa Ora -The Centre for Health with oversight by Māori leadership. This method therefore supports Māori values and practices over-and-above Western forms of research interviews (Lord et al., 2022).

Three, hui took place which and consisted of an interactive workshop format with group interview. Sleep-related information was shared with pauses for feedback and kōrero (disucssions) from participants with regards to their own experiences. Topics included the functions of sleep; defining the regulation and patterning of sleep; factors affecting sleep health across the lifespan; and strategies for managing problematic sleep. Open-ended questions related to these areas with the freedom for participants to reflect on the topics with regards to norms, beliefs, and practices relevant to them, their whānau and te ao Māori (the Māori world). Hui were hosted within the familiar space of Manawa Ora. They were led by a representative of the center who was well known to the participants and competent in te reo me ona tikanga Māori (Māori language and customs) alongside the lead sleep researcher. In concordance with the kaupapa (policy) of the center, kai (food) was prepared and shared prior to each hui, and time was allocated for pepeha and whanaungatanga (introductions and relationship forming). In concordance with the center's tikanga, comments were recorded by note taking and oral clarification (as opposed to audio recording). Participants also had the opportunity to write notes during or contact the research team afterwards to offer further insights which may not have occurred to them at the time, or they didn't feel comfortable raising in the group environment. However, other than expressions of gratitude for the sessions, no further information was offered in this way. Finally, participants were provided a $20 koha (gift) in appreciation of their contributions.

### 2.2 Participants

Aging Well through Eating, Sleeping, Socializing and Mobility (AWESSoM) represents a NZ National Science Challenge program of research integrating multiple projects across population groups which had the overarching aim to maximize independence and push back the threshold of disability (Lord et al., 2022). The Ngā Pou o Rongo (Māori wellbeing in aging) study within AWESSoM was led by Manawa Ora - The Centre for Health in Tauranga. The purpose of was to deliver a 12-week kaupapa Māori (Māori knowledge, methods and philosophy) based lifestyle program to whānau that allows participants to imbed their cultural context in a manner that is balanced with the medical and scientific aspects of care for long-term condition management and aging. The program was led by whānau and based on interpretations of hauora (health & wellbeing) and incorporates medical, exercise physiology and nutrition science in balance with mātauranga Māori (Rochford, 2004). This expands upon previous research that focused on a similar approach for the reduction of cardiovascular risk among older Māori and their whānau (Rolleston et al., 2017).

Older Māori (aged over 60 years) deemed at risk of cardiovascular disease and their whānau were included in the AWESSoM Ngā Pou o Rongo project (Lord et al., 2022). At the time of sleep study preparations, 25 participants were engaged with the Ngā Pou o Rongo project (including 12 older Māori). These participants were informed of this adjunct sleep project by email and spoken advertisement through Manawa Ora, The Centre for Health. This project was approved by The Southern Health and Disability Ethics Committee, New Zealand. Twelve participants took part across three stand-alone hui (60–90 min duration, 3–5 participants per hui). The majority (10) of participants identified as Māori, and were women (9), ages ranging from 47 to 72 with the majority over 60 years of age.

### 2.3 Analysis

The approach to analysis bridged between mātauranga Māori concepts of wellbeing (Durie, 1985; Valentine et al., 2017) and Western social science where the embodiment of sleep is considered as a visceral and experiential phenomenon alongside social norms and practicalities (Sokolowski, 2000; Meadows, 2005). Notes were collated and compared immediately after the hui. A data-driven thematic analysis of the notes was conducted before review and development by the wider research team. This approach allowed for initial descriptive data and codes to aid the construction of basic themes (Braun and Clarke, 2019). Themes underwent further reflexive discussions and revisions and illustrative quotes identified, allowing for the organization of data into broader social-cultural representations of sleep with aging.

## 3 Findings

Four key themes were constructed that represent the key kōrero (discussions) and whakaaro (thoughts). These concerned the conceptualizing of sleep; changing commitments around sleep and wake; and barriers and facilitators for supporting sleep. These are described below and illustrated using noted quotes.

### 3.1 Conceptualizing sleep

Physiological explanations of sleep regulation and architecture were generally accepted and the functions of sleep for physical and mental health acknowledged. Some reflected on how the state they felt they were currently in (regarding their limited physical and/or mental health) may be associated to long-term but small sleep-related sacrifices:

*I'm aware of all those properties and, if I slept well, I could perhaps achieve them. But I get by*.

Some reported using technology to measure sleep to visualize and compare recordings between days and to the software-defined norms to help understand their waking health:

*The Fitbit was useful as I had a feeling sleep was bad and that explains how I am now*.

However, sleep was also considered as requiring broader lenses for a complete conceptualization as well as its relationship to the realms of waking and spiritual life. The structure and experience of sleep as well as periods of sleeplessness were considered to align with wellness associated with whānau or whakapapa (genealogy), for example through dream experiences or wairua (a spirituality):

*Sleep provides the opportunity to experience something else the mental and spiritual release of dreaming, however good or crazy*.

Similarly, the wairua or mauri (life force) of an environment or space was also presented as influencing sleep. Such as sleeping on the marae (traditional Māori building complex for gatherings) for events such as tangihanga (funeral processes) which was noted as providing opportunity for spiritual wellbeing:

*It's our Wairua, our spiritual world. Wairua particularly around the time of tangi. If there is a tangi going on I can feel their wairua around me and that keeps me awake during that phase [and that's ok]*.

Some recalled experiences of increased wakefulness in the night and early morning compared to when they were younger. Such periods were also deemed to provide positive opportunities for connection from a wairua perspective. However, some anxiety was described in relation to how much sleep they thought they should be achieving or what else they could be achieving with the time:

*I can feel my heart popping through my chest – anxious about not waking up on time and then end up tired in the meeting*.

*My brain is so active at night and my thought patterns so clear so I might as well work at night*.

Finally, sleep was metaphorically referred to as the passive offline state of death as a justification for embracing periods of wakefulness in older age:

*The really big sleep is coming, so I may as well stay awake*.

### 3.2 Changing commitments around sleep and wake

Many comments concerned the shifting commitments that had come with advancing age and retirement. For some, former work life with its long or antisocial hours was reported to have had a lasting impact on sleep. Considerable time was reportedly required to adjust to a new state of normal where a more relaxed lifestyle was anticipated (yet sleep was still elusive):

*I've slept 2 h a night all my life now… I'm retired now – how do I sleep I know I'm meant to?!*

*Work schedules played a big role, they call it 9-5 but mine was more like 8-7. It governs your life for a long time. Now I'm in a new zone, don't have to get out of bed – “every day is a Sunday,” it's a major change - it took 2–3 years to adjust to the new lifestyle*.

However, most reports concerned the adaptation from the tight schedules of organized work to feeling busier and a need to be available on a more constant basis during older adulthood:

*There's too much to do so I just take it out of my sleep!*

This included sharing of the night duties associated within the whānau such as bedsharing and soothing of mokopuna (grandchildren). As well as commitments as an older member of the community and as service to whānau, hapū (subtribes), and iwi (tribes). Such commitments were reported as being less schedular than those of their former working life and provided opportunity for alignment with tikanga Māori and whānau needs Therefore, some had become potentially very busy across all hours of the day. However, this was also described as a natural progression and an honorable aspect of aging:

*[We are] giving back to our people as we have the time now, it's a form of responsibility and we didn't have time before as working*.

*There's no excuses as no time schedules like for work. Work was structured while this mahi is more fulfilling but scattered across the day and night*.

Many identified that they could catch up on sleep when time allowed. Although also commented on the pace of life leading them to feel very busy which subsequently impacts on their ability to relax and sleep and at their preferred times:

*I haven't stopped since I finished [working]*.

### 3.3 Barriers and facilitators for supporting sleep

An overarching appreciation for sleep was demonstrated alongside acknowledgment of the challenges to support good sleep habits:

*We all know sleep is important, we just don't prioritize it as much as we should*.

Participant's current sleep experiences were often described or justified through stories comparing sleep to how it used to be when they were younger.

*I used to think I loved my sleep, deep sleep…what's changed!?*

Sleep was also framed in respect to how other generations previously or currently slept:

*I was out chopping wood with my mate, stacking it, putting it away and that, I said “where are the kids?” he said “sleeping,” I said ‘at two-o'clock in the afternoon?!, they should be out here!*

Barriers and enablers of sleep appeared to be well known. Individual sleep tips were shared and compared concerning the regulation of sleep, behaviors around bedtime, and relaxation.

Those who had experienced a slower pace of life with retirement often presented day sleeping as a useful tactic as they had “nothing else to do” or fewer responsibilities:

*I'm not tied to someone else's schedule – as long as I can do for [partner/care recipient] what he needs. If there's a [sleep] imbalance you have to catch up somewhere*.

*We nap, mostly after lunch—we allow ourselves to do that we don't have little children, it's alright, because we don't sleep well at night*.

This ability to nap was presented as novel with retirement despite acknowledgment that such practices are normal in other cultures as well a prolific behavior of the teenagers within their whānau who “sleep the day away” while the adults completed necessary domestic duties. Napping was not something that the participants felt would have been socially accepted when they were younger or in paid employment despite their awareness that it could have served them well:

*You're not allowed to nap at work—Don't feel allowed to sneak off to nap at work as I might be considered failing and old. But if I could have snuck off for 5–10 min, I'd have felt much better*.

Periods of social disconnect such as being off grid and amongst nature were used as examples of providing superior sleep:

*Whenever we go to X—there's less reception and we go to sleep when the sun sets and so I sleep really well. And I wake up feeling refreshed*.

Such examples were juxtaposed against what was considered as socially “normal” and infer the inability to be able to achieve refreshing sleep when in the typical busy or urban environment.

Relaxation tips included typical ideals around caffeine, alcohol, and screen use at bedtime. Rongoā (traditional Māori healing methods) such as thermal baths, mirimiri (massage) and breathing exercises were also highlighted as aiding good sleep. For example:

*I sleep better after mirimiri as I can still feel that rub…Thermal baths have the same affect going home, completely relaxed us—that's what we grew up on… the heat helps with sleep*.

Reflections on the sleep of others were provided as examples of both helping and hindering sleep. For example, the movements, wakefulness, or care needs of a bed partner:

*Movement in the bed is a big issue having two separate beds but together—I find that brilliant because [otherwise] I can feel him moving around and he can feel me moving around…movement is a sleep killer*.

*I struggle to get to sleep then husband is there “pushing up the Z's”—it just makes me so angry!*

*At first [after husband became in need of care] I wouldn't sleep as checking to see if he was breathing*.

Some participants described the challenges of living in a multi-generational whare (house) with shared sleeping spaces with mokopuna:

*Moko shares the bed with me every night, that's just the way it has to be*.

While this was presented as somewhat disruptive to sleep, the role of looking after mokopuna was also described as having an overarching calming effect. Similarly, the positive social connections concerning communal sleeping were common:

*I have the best sleeps on the marae, especially when there are a lot of people around…quiet talking in the background. Someone else is awake, to protect you*.

*Sleep can be facilitated if surround yourself with positive people—good kōrero, fulfillment, relaxing*.

Such accounts indicated that how, even if in a noisy environment or away from the typical bed, sleeping communally with whānau or noho marae (staying on marae) for events such as tangihanga was also considered as facilitating sleep alongside feelings of comfort and safety, alongside cultural connection.

## 4 Discussion

This study describes the nature of sleep among older Māori and their whānau engaged with a health program. Findings from the hui reflected multifaceted concepts of the meaning and purpose of sleep as well as recognition of sleep-related changes with aging and factors facilitating “good” sleep. Such findings align with the holistic nature of the Māori worldview and approach to health (Durie, 1985; Valentine et al., 2017) and therefore do not simply reflect individual experiences but also link to scientific, social, and cultural understandings of sleep and aging that help inform and interpret sleep-related experiences.

Previous epidemiological studies (including from NZ) signal that, despite well-recognized physiological and pathological threats to sleep health in later life (Edwards et al., 2010), there appears to be a plateauing in the proportion of self-reported sleep problems with advancing age. Ethnic disparities of sleep health also appear less apparent among older adults (compared to younger adults) (Grandner et al., 2012; Gibson et al., 2016, 2020; Paine and Gander, 2016). Previous interviews with older New Zealanders indicated a resistance to pathologizing sleep grounded in a tendency to normalize the deterioration of sleep with aging, plus beliefs that little could be done to resolve sleep problems (Crestani et al., 2022). Some older Māori also suggested that the quiet night and periods of sleeplessness could provide positive opportunities such as reflection or connection with wairua or ancestors (Crestani et al., 2022). The current study expands on these findings using group interviews with predominantly older Māori with symptoms of cardiovascular health issues. Themes concerning sleep changes and facilitators were presented using common themes around the rewarding nature of waking life with older age as well as the acknowledgment of somatic, environmental, community, and spiritual dimensions which all contributed to experiences of both sleep and aging.

Western sleep science has been predominantly based in medical models. These foster physiological approaches for defining sleep and its disorders as well as pharmacological treatments and behavioral interventions (Dement, 2008; Williams, 2008; Barbee et al., 2018). Such models have also informed “sleep hygiene” guidelines for optimizing sleep alongside general health and waking function. In the present study, sleep was acknowledged as being (in part) of the body with its physiological functions for health and wellbeing. An awareness around the implications of sleep's purpose for wellbeing reflects the growing field of sleep science and heightened public knowledge and commercialization of sleep and it's monitoring (Coveney et al., 2023). Some participants referred to using tracking devices to help them quantify or visualize their sleep timing and patterning. Such activities are increasingly common in modern society as a plethora of “smart” devices and health apps are available (Coveney et al., 2023; Kubicki et al., 2023). Sleep tracking has a place for comparing sleep across time and identifying markers of clinical sleep issues. However, can also have paradoxical effects if data are incessantly compared to software norms or social ideals (Baron et al., 2017; Coveney et al., 2023; Kubicki et al., 2023). Here, tracking was referred to as a useful tactic to justify feelings of poor sleep and waking fatigue as opposed to avid surveillance. While sleep was described in this visceral form, participants also embodied sleep as a spiritual experience.

Spiritual interpretations of sleep are seldom included in contemporary Western models despite some sleep-related experiences (such as sleep paralysis and unique dream states) being historically described as spiritual phenomena (Carvalho et al., 2016). However, for Māori, wairua is a vital dimension of the lived experience which is interwoven with the individual, their actions, and environment (Marsden, 2003) all of which are essential to the existence and wellbeing of the physical body (Durie, 1985; Henare, 2001). The role of wairua in sleep is derived from mātauranga Māori knowledge systems. These incorporate cosmological, epistemological, and ontological knowledge which are relevant to both the material and spiritual realms (Marsden, 2003). Humans are considered a consequence of such dimensions intertwining and the ability to traverse between realms is more widely accepted than in Western cultures. Therefore, during sleep the physical body can be considered as resting in the material realm while the wairua awakens and moves through various metaphysical dimensions relevant to te ao Māori (Valentine et al., 2017). Such ideas are apparent in the present research through examples of sleep providing connection with meaningful environments or ancestry as well as feelings of physical and cultural security. Such experiences were typically provided through sleeping in unique places or communally, especially during exceptional times of grief such as tangihanga. Previous works explain such concepts with ideas around sleep facilitating the disembodiment of the wairua so that it can travel and interact with those of others (both living and deceased). During sleep, the wairua may also acquire knowledge to support the physical self in safely navigating the waking, material realm as well as guard the physical body during the vulnerable state of sleep (Best, 1900; Lindsay et al., 2020). The present findings expand on this with empirical research. Positive aspects of sleep practices were highlighted with regards to wairua and broader cultural connection. Furthermore, periods of sleeplessness which are stereotypically considered negative or problematic were not always considered as such by these participants—providing an example as to how the sensitivity and context for problematizing sleep can differ. This research then, challenges standard medical ideas around what sleep is and its functions as well as providing a greater appreciation for what “normal” or “problematic” sleep is beyond Western models typically used in academic and clinical practice. Therefore in conceptualizing sleep, it is important to consider the individual and collective spiritual experience as well as the environmental and social context of sleep arrangements.

The timing of sleep is often considered to be patterned around the schedules of work with long hours or shift work having greater influence over sleep's regulation (Williams, 2011; Wolf-Meyer, 2019). Indicators of sleep problems have been associated with earlier retirement from paid work (Myllyntausta et al., 2021). Furthermore, there are common assumptions that individuals will have more autonomy over their sleep and be able to luxuriate with better sleep post-retirement. However, previous research indicates that negative impacts of former work schedules can be long lasting for sleep timing and health (Mori et al., 2005; Myllyntausta et al., 2019). Indeed, Crestani et al. (2022) interviews indicated that New Zealanders who had previously worked shift patterns found it difficult to adjust to sleeping within “normal” time frames that aligned with others and with what they expected from being work-free. This is particularly relevant, given Māori have been identified as of increased likelihood to work shift patterns compared to non-Māori (Paine and Gander, 2016). Such themes were reiterated in the present research. For example, participants noted how they would wake inconveniently early in the morning, as per their former work schedules.

While opportunities to recoup lost sleep were noted (such as with rest or naps), participants overwhelmingly noted an increase in activities and responsibilities with older age. Previous research using European data illustrates shifts in activities indicative of “doing ageing” after ceasing work (Wanka, 2020). Activities which predominantly replaced work time included personal activities (such as eating, sleeping, and personal hygiene); household chores (including childcare, cooking, gardening, and shopping); and media use (including reading, watching television, or surfing the internet). These activities each accounted for an additional 1.1–1.8 h compared to working adults. However, social (including cultural) activities only increased by an average of 30 min per day. Conversely, the present interviewees spoke predominantly of increased time allocated to family and extended whānau care as well as duties related to their position as an older member of the iwi particularly concerning cultural practice and ceremony. Unlike the schedules of paid work or more organized hobbies and clubs typical of European models of retirement, the commitments described here had little-to-no schedule. Therefore, a feeling of being “busier than ever” was reported. Yet such activities were also described as more rewarding that those of employed work. This was because they often involved commitments to whānau and iwi which were described as honorable with regards to age and stage. Such comments are indicative of the unique social and cultural priorities of older Māori (McDonald, 2016; Rolleston et al., 2022) as well as the collectivist approaches more apparent in Indigenous cultures, where sleep is more likely to be patterned according to cultural norms and community wellbeing rather than an individual's needs or preferences (Jones et al., 2017). Here, the arrangements and timing of sleep were often communal in nature, particularly in relation to supporting extended family and child rearing. Māori as well as Indigenous Aboriginal and South Americans have been observed to align more so with concepts of “event time” rather than “clock time” compared to non-Indigenous cultures. As such, activities across the day (and night) are more likely to be informed by communal and environmental relationships and obligations, as opposed to being rationalized by imperial time cues (Janca and Bullen, 2003; Lo and Houkamau, 2012). The present research exampled how sleep is often patterned around that of others. Furthermore, sleep and dreaming were alluded as providing opportunity for communal, creative, or spiritual experiences broadening the conception and functions of sleep beyond physiological and medical models that have dominated much modern sleep science.

Finally, sleep was situated within the roles and responsibilities of older adulthood. For Māori, older age is typically considered with greater value and community purpose compared to Western connotations of age-related hardship, loneliness, and illness (Waldon, 2003; McDonald, 2016). Furthermore, cultural engagement has been identified as independently related to health outcomes among older Māori (Dyall et al., 2014). Such ideas were reflected in the present research with reports of gratitude related to living a long life and the opportunities that both waking and sleeping life bring. This included desires to stay alert, socially engaged and productive while able. This was embedded in reflections on advancing age encompassing an expected decline in health and ultimately a ceasing of production and life itself.

### 4.1 Considerations

When considering these findings, it is important to appreciate the research limitations. By involving participants already enrolled in a whānau health program and incorporating elements of a sleep workshop, participants may have been primed regarding how they reported on sleep. To counter this, the group interviews were constructed to be a relaxed non-judgmental hui-like atmosphere where participants could feel comfortable among those close to them and to report experiences in an unbiased form using confidential recordings. Note-taking was used opposed to digital voice recordings. While this limited the ability to fully transcribe and analyze discussions statement-by-statement, this practice was in alignment with those of the wider Ngā Pou o Rongo project and Manawa Ora, Centre for Health. It also encouraged the researchers to fully engage with the hui and use reflexive forms of conversation to verify what was being said with participants in real-time with regards to representation. Furthermore, the collation and comparison of notes immediately after the hui began the process of coding and subsequent thematic construction and interpretation This enabled confirmability and transparency between the research team, the participants and the data.

The study included a sample who lived in the same region and were mostly known to each another through their whānau-based healthcare provider. The themes corroborate and expand upon previous works involving one-on-one interviews with healthy older Māori from elsewhere (Crestani et al., 2022). However, are not transferable to all contexts. For example, those with severe physical or mental health conditions, or of limited capacity because of their living or caregiving situations are underrepresented. Such populations have been identified as having more extreme sleep disruptions (Bliwise, 2016; Miner and Kryger, 2020). However, tendencies to normalize such disruptions are also apparent in such situations (Gibson et al., 2023b) and reflect broader social discourses of aging and disease (Breheny et al., 2023). The inclusion of those known to one another is also considered a strength with regards to forming a clearer understandings and representation of sleep as a social, familial and cultural construct with regards to modern NZ society.

### 4.2 Conclusion

The present findings add to the growing literature concerning social-cultural beliefs, attitudes, and practices concerning sleep during an era when sleep is increasingly commodified and public health messages are grounded in Western ideals. While epidemiological works have incorporated self-reported sleep problems among Māori, this is among the first qualitative sleep studies concerning older Māori. The hui elucidated unique social and cultural conceptions used for defining and supporting sleep. Cultural and social practices as well as spiritual explanations were often used to justify sleep patterning and arrangements. Such concepts also provided novel (within Western science at least) interpretations of experiences of sleep, dreaming, and periods of sleeplessness. Sleep was acknowledged as functional for supporting physical and mental health, and participants had an awareness of methods useful for supporting “good sleep.” However, sleep was overwhelmingly positioned as fitting in alongside the busy nature of waking community life and the responsibilities that come with being of older age within Māori culture.

## Data availability statement

The datasets presented in this article are not readily available because the datasets generated and analyzed for this study are considered property of the participants and is not freely available. Materials may be available upon request to interested researchers via application to Manawa Ora The Centre for Health. Requests to access the datasets should be directed to AR, anna@thecentreforhealth.co.nz.

## Ethics statement

The studies involving humans were approved by the Southern Health and Disability Ethics Committee, New Zealand. The studies were conducted in accordance with the local legislation and institutional requirements. The participants provided their written informed consent to participate in this study.

## Author contributions

RG: Writing – review & editing, Writing – original draft, Project administration, Methodology, Funding acquisition, Formal analysis, Conceptualization. HL: Writing – review & editing, Project administration, Methodology, Formal analysis, Conceptualization. EK: Writing – review & editing, Visualization, Supervision. AR: Visualization, Resources, Writing – review & editing.

## Figures and Tables

**Table:** 

Hapū	Subtribe
Hauora	Health and wellbeing
Hui	Group meetings and discussions
Iwi	Tribe
Kai	Food
Kaupapa	Policy
Kaupapa Māori	Māori knowledge, methods and philosophy
Koha	Gift
Kōrero	Discussions
Māori	Indigenous people of NZ
Marae	Traditional Māori building complex for gatherings
Mātauranga Māori	Māori knowledge
Mauri	Life force
Mirimiri	Massage
Mokopuna	Grandchildren
Noho marae	Staying on marae
Pepeha	Introductions
Rongoā	Traditional Māori healing practices
Taha wairua	Spiritual health
Taha hinengaro	Mental health
Taha tinana	Physical health
Te Ao Māori	The Māori world
Te reo me ona tikanga Māori	Māori language and customs
Tikanga	Correct procedures, customs
Wairua	Spirituality
Whakaaro	Thoughts
Whakapapa	Genealogy
Whānau	Extended family
Whanaungatanga	Relationship forming
Whare	House
Whenua	Land

## Glossary

This glossary is provided to inform the reader of some of the basic Māori concepts used within this journal article. It is acknowledged that Māori words often have varied meanings and are complex and as such this glossary captures the meanings of the words as they relate to this article. For further information on translations see Moorfield (2011). *Te aka: Māori-English, English-Māori dictionary and index (New expanded ed)*. Pearson.
